# Surgical treatment strategies for extra-pelvic intravenous leiomyomatosis

**DOI:** 10.1186/s13023-020-01394-9

**Published:** 2020-06-16

**Authors:** Hua Li, Jing Xu, Qiaowei Lin, Yong Zhang, Yun Zhao, Hanxing Tong, Ruiqin Tu, Demin Xu, Chunsheng Wang, Weiqi Lu

**Affiliations:** 1 Department of Cardiac Surgery, Zhongshan Hospital, Fudan University, No. 180 Fenglin Road, Shanghai, 200032 China; 2 Department of General Surgery, Zhongshan Hospital, Fudan University, No. 180 Fenglin Road, Shanghai, 200032 China; 3 Department of Obstetrics and Gynecology, Zhongshan Hospital, Fudan University, Shanghai, China

**Keywords:** Intravenous leiomyomatosis, Intracardiac leiomyomatosis, Single-stage surgery, Surgical strategies, Vena cava invasion

## Abstract

**Background:**

Extra-pelvic intravenous leiomyomatosis (IVL) extending into inferior vena cava (IVC) or heart (i.e. intracardiac leiomyomatosis, ICL) is an extremely rare benign disease. No consensus has been reached on the optimal surgical strategy. The aim of this study is to introduce four types of one-stage surgical strategies including less invasive options and a guideline to select patient-specific strategy for this disease.

**Methods:**

Twenty-four patients of extra-pelvic IVLs receiving one-stage resections at the Zhongshan Hospital from July 2011 to November 2019 were reviewed retrospectively. Base on the initial experiences of the indiscriminate choices of tumor thrombectomies through sterno-laparotomy under cardiopulmonary bypass (CPB) in 6 ICLs, an anatomy-based guideline for four types of surgical strategies was developed and applied for the next 18 patients.

**Results:**

Under the direction of guideline, tumor thrombectomies through single laparotomy were applied without CPB in 2 ICLs and 4 IVLs confined in IVC, or with CPB in 7 ICLs. Guideline-directed double-incisions with CPB were applied in only 5 ICLs, including 1 receiving mini-thoracotomy and 4 receiving sternotomy because of tumor adherences with right atriums in 2 and with pulmonary arteries in 2. All 24 patients accomplished one-stage panhysterectomy, bilateral adnexectomy and complete resections of intracaval and intracardiac tumors. For residual pelvic intravenous tumors in 19 patients, 17 received macroscopically complete resections while the other 2 failed because of high risk of hemorrhage. Intraoperative blood losses, operation time and hospitalization expense in the single-laparotomy non-CPB group were significantly lesser than the other groups. In CPB groups, inpatient stay and hospitalization expense in the single-incision group were significantly lesser than the double-incisions group. All patients were alive and free of recurrences during a mean follow-up of 35.4 ± 27.2 months (range, 1–100 months). The pelvic tumor residues in 2 patients remained unchanged for 51 and 52 months since operation, respectively.

**Conclusions:**

For various extra-pelvic IVLs, the 4 types of surgical strategies including less invasive options are feasible, providing these are selected by a guideline base on the tumor extension and morphology. The proposed guideline is believed to accommodate more patients receiving less invasive surgery without compromising the curative effect.

## Introduction

Intravenous leiomyomatosis (IVL) is a rare benign neoplasm of smooth muscle origin. The primary lesion is typically in the uterus and extends to extrauterine venous circulation. Most of IVLs were premenopausal women. The probable etiology is that IVL originates from the neoplastic smooth muscle cells which intrude the veins of the genital system [1] or proliferation of smooth muscle cells of the vascular tunica media [2, 3]. In IVL, the expression of *HMGA2* (locus 12q14.3) might be associated with chromosomal rearrangement [4], the loss of 22q12.3-q13.1 and absence of G > A transition at nucleotides c.130 or c.131 were associated with a high level of genetic instability [5]. Less frequently, IVLs can extend outside of pelvic cavity into the inferior vena cava (IVC) or cardiac chambers (i.e. intracardiac leiomyomatosis, ICL). Since the first report of IVL extending into heart in year 1907 [6], there were only approximately 300 cases of extra-pelvic IVLs reported in the literatures [1, 7, 8], most by case reports. Because IVLs were often asymptomatic until entering heart, most of the patients presenting in clinics were ICLs. If untreated, tumor extension inside circulatory system could cause Budd-Chiari syndrome [9], obstruction of the right heart [10, 11], pulmonary embolism [12, 13] and sudden death [11, 14]. The best treatment for extra-pelvic IVL is radical surgery, and no tumor recurrence was reported after the complete resection of the tumor within the circulation system combined with panhysterectomy and bilateral adnexectomy [7, 8, 15–18]. The longest postoperative follow-up was 12 years. Recurrence occurred in 1/3 patients following incomplete resections after short follow-up periods [7]. Four out of 7 patients who refused panhysterectomy and bilateral adnexectomy developed recurrence [8].

Since the first complete resection of ICL [19] through a two-stage approach in year 1982, numerous surgical strategies with great discrepancies have been introduced among various surgical teams. For the ICL, the initial approaches of staging sternotomy and laparotomy [16, 19, 20] had changed to one-stage procedure [7]. In most reports [7, 15, 21, 22], one-stage resection of ICL utilized sterno-laparotomies, cardiopulmonary bypass (CPB) and even circulatory arrest. For certain types of ICL, several less invasive strategies have been proposed in individual cases, such as simple abdomen incision [23–27], avoidance of CPB [23, 25–27] or endoscopic surgery [28]. Due to the extreme paucity and wide heterogeneity of extra-pelvic IVL, no consensus has been reached on the optimal surgical strategy.

It is noteworthy that the intraluminal tumor tissue was firm enough to endure high tensile strength without breaking [24]. Also, this tumor is covered by a layer of endothelial cells without superficial clots [18]. If the tumor doesn’t compress the surrounding endothelial surface, which may cause circular adherence, it will be freely movable within the caval or cardiac chamber. These unique features imply the feasibility of tumor thrombectomy by extraction of the remote part of this linear tumor. Base on these ideas, the present study developed some modified surgical strategies and tries to classify anatomic features guiding surgical planning.

## Patients and methods

### Patients

Twenty-four consecutive patients receiving one-stage resections of extra-pelvic IVLs at the Zhongshan Hospital from July 2011 to November 2019 were retrospectively reviewed. This study was approved by the ethics committees of Zhongshan Hospital, affiliated with Fudan University. Informed consent was signed by each patient.

### Surgical planning for tumor Thrombectomy from IVC and heart

All patients received careful preoperative morphologic assessment. Diameters of intracaval tumor and carrying vein were obtained by computed tomography venography or three dimensional reconstruction image. Diameter and mobility of intracardiac tumor were obtained by echocardiography.

**Fig. 1:**
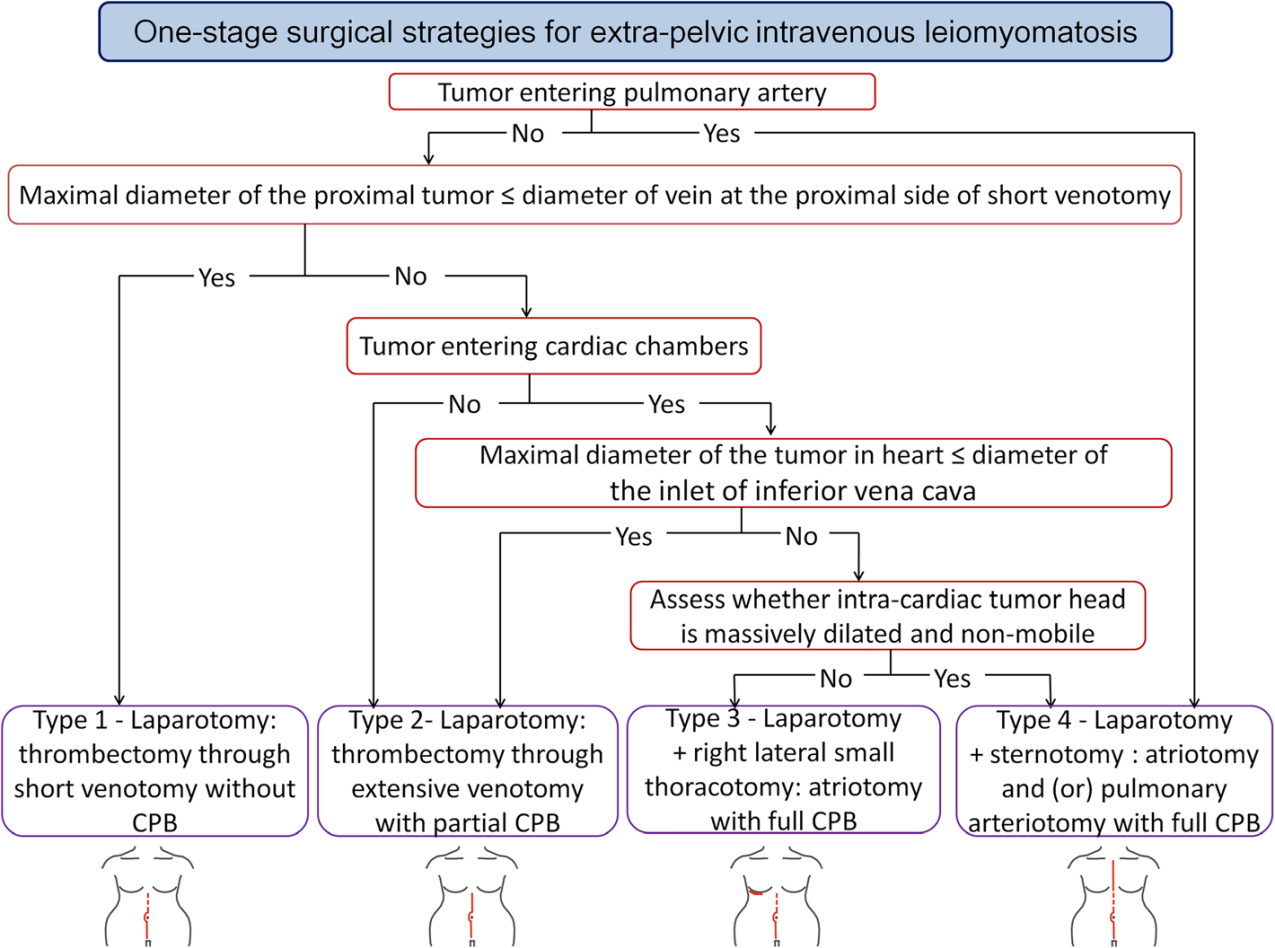
Guideline of one-stage surgical strategies for extra-pelvic intravenous leiomyomatosis. CPB, cardiopulmonary bypass

**Fig. 2:**
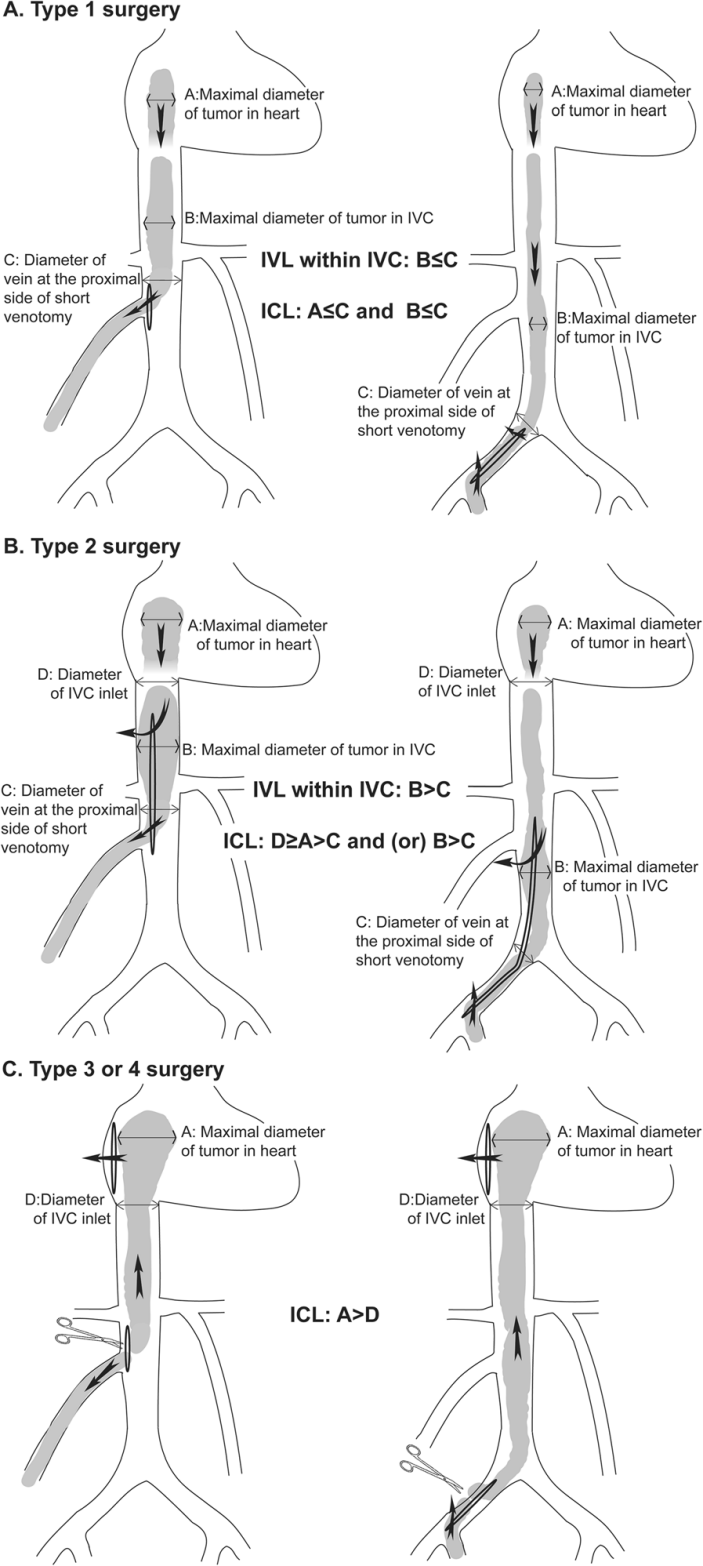
Schematic diagram and selecting criteria of 4 types of surgeries. IVC, inferior vena cava

By a multidisciplinary team, all patients were planned for one-stage resections of tumors inside circular system, panhysterectomy and bilateral adnexectomy. For the first 6 patients with ICLs, indiscriminate choices of sterno-laparotomies were planned. Base on the experience of these 6 cases, modified less invasive surgical strategies were pursued for the next 18 patients. Surgical strategies were classified into 4 types ranked in order of the extent of surgery. Patient-specific strategy was selected by a guideline which was a stepwise integrated approach according to tumor extension and morphology (Fig. 1). Type 1 ~ 2 surgeries needed only laparotomy without chest opening. Type 1 surgery (Fig. 2a) represented tumor thrombectomy through small venotomy at the IVC proximal to the ovarian vein or at the common iliac vein proximal to the internal iliac vein. Cardiopulmonary bypass (CPB) was not needed. Type 2 surgery (Fig. 2b) represented tumor thrombectomy through an extensive venotomy aided by partial CPB. Type 3 ~ 4 surgeries (Fig. 2c) needed double-incisions and full CPB to perform atriotomy and venotomy. Firstly, tumor was transected through a venotomy within abdomen. Then, the proximal part of tumor was pulled upward out of atriotomy and the distal part of tumor was removed through that venotomy. With regard to the approach of chest-opening, type 3 surgery only needed a mini-thoracotomy in the right fourth intercostal space, while type 4 surgery needed sternotomy. The abdominal midline incision started at symphysis pubis and, depending on the site of venotomy, extended to above the umbilicus or the xiphoid (Fig. 1). Partial CPB was set up through cannulation of femoral artery and vein, while full CPB needed another venous drainage through cannulation of superior vena cava, heart remained beating. Mild hypothermia(33–35 °C) was applied.

### Surgical procedures after tumor Thrombectomy from IVC

As shown in Fig. 2, if the ovarian vein was the path of tumor, the distal length of IVL was resected with the ovarian vein; if the IVL entered IVC through internal iliac vein, the distal part of tumor was dragged out or transected under the inlet of internal iliac vein. Bilateral adnexectomy and panhysterectomy (or resections of residual hystera and adnexa uteri after previous gynecological operation) were planned for all. Resection of residual pelvic intravenous tumor was also planned. The asymptomatic pulmonary benign metastasizing leiomyomas were not considered for surgical resections.

### Follow-up

Patients were followed up every 3 months for the first year, then every 6 months for the next 3 years, then every 1 year afterward. Tumor recurrence was considered to be the appearance of a new mass or enlargement of tumor residue.

### Statistical analysis

Values for the count of clinical data are expressed as mean ± standard deviation (SD) or as n and percentage. All analyses were performed using SPSS 20. A Levene’s test for homogeneity of variances was used to check for equal variance. The 1-way ANOVA and post-hoc LSD t-tests were used for comparison among groups with equal variance; meanwhile, Kruskal-Wallis with Dunn’s post-hoc test with Bonferroni correction were used with unequal variance. Statistical significance was set at *P* < 0.05.

## Results

### Patients characteristics

As shown in Table 1, all 24 patients were female with ages ranging from 34 to 62 years (mean 44 ± 6.9 years). At present, 4 IVLs confined in IVC and 6 ICLs were asymptomatic, the symptoms of the other 14 ICLs included dyspnea in 6, syncope in 2, abdominal distension in 2, lower limb edema in 2, cough in 1, anorexia in 1, vaginal bleeding in 1, and NYHA functional class III or IV in 3. Among 13 patients (54.2%) with a history of uterine fibroid, 12 received previous gynecologic operations including hysteromyomectomy in 5, panhysterectomy in 3, subtotal hysterectomy in 2, adnexectomy in 2. The right internal iliac vein was the most frequent path where tumor entered IVC (14 cases, 58.3%), followed by the right ovarian vein (9 cases, 37.5%). In all patients, diagnose of IVL was made preoperatively and confirmed by pathology. Pulmonary nodules in 3 patients were diagnosed preoperatively as benign metastasizing leiomyoma by positron emission tomography.

**Table 1:** Patient characteristics and treatment courses of 24 patients with extra-pelvic intravenous leiomyomatosis

No.	Age	Main symptoms	Path of tumor	Extension	Surgical procedure	Site of extra-pelvic tumor adherence	Follow-up (mon)
1	41	Abdominal distension	Right IIV	Right ventricle	Sternolaparotomy	Right atrium	100
2	34	None	Bilateral IIV	Right atrium	Sternolaparotomy	–	99
3	49	Lower limb edema	Right IIV	Right atrium	Sternolaparotomy	Infra renal IVC	68
4	48	None	Right IIV	Right atrium	Sternolaparotomy	–	64
5	50	Abdominal distension	Right IIV, right ovarian vein	Right atrium	Sternolaparotomy^a^	–	52
6	37	Vaginal bleeding	Right IIV, right ovarian vein	Right atrium	Sternolaparotomy^a^	Right atrium	51
7	42	None	Right ovarian vein	Supra renal vein	Type 1 surgery	–	49
8	62	Anorexia	Right ovarian vein	Right ventricle	Type 2 surgery	The inlet of right ovarian vein	47
9	47	Dyspnea	Left ovarian vein	Right atrium	Type 4 surgery	Right atrium	41
10	30	None	Right IIV, right ovarian vein	Right atrium	Type 2 surgery	–	37
11	50	None	Right IIV	Right ventricle	Type 2 surgery	–	32
12	44	None	Left ovarian vein	Right atrium	Type 2 surgery	–	32
13	55	Lower limb edema	Right IIV	Right atrium	Type 2 surgery	Infra hepatic IVC	27
14	44	Syncope	Right ovarian vein	Right atrium	Type 4 surgery	Right atrium	27
15	47	Dyspnea	Left IIV	PA^b^	Type 4 surgery	PA and left CIV	26
16	45	Dyspnea	Right IIV, right ovarian vein	Right ventricle	Type 2 surgery	Right CIV	17
17	45	Dyspnea, cough	Right IIV	PA	Type 4 surgery	PA	17
18	34	Syncope	Right IIV	Right ventricle	Type 2 surgery	–	16
19	41	None	Right ovarian vein	Supra renal vein	Type 1 surgery	–	16
20	43	Dyspnea	Right IIV	Right atrium	Type 3 surgery	Infra renal IVC	15
21	44	None	Right ovarian vein	Secondary porta of liver	Type 1 surgery	–	7
22	46	None	Bilateral IIV	Right atrium^c^	Type 1 surgery	–	6
23	39	None	Left IIV	Secondary porta of liver^d^	Type 1 surgery	–	2
24	47	Dyspnea	Left IIV	Right atrium	Type 1 surgery	Left CIV	1

^a^ Incomplete resection of pelvic intravenous tumor

^b^ Multiple nodules up to 1.9 cm in diameter in the middle lobe of right lung

^c^ Solitary nodule of 1.4 cm in diameter in the inferior lobe of right lung

^d^ Multiple nodules up to 1.5 cm in diameter in the both lungs

*CIV* common iliac vein; *IIV* internal iliac vein; *IVC* inferior vena cava; *PA* pulmonary artery

### Indiscriminate sterno-laparotomies for the first 6 ICLs

For them, full CPB was set up, the tumors were dragged upward through atriotomy. By such strategy, only 2 cases accomplished totals removal of intracaval tumor. In the other 4 cases, additional venotomies at common iliac vein or IVC were performed to remove tumor residues.

### Guideline-directed surgical procedures for the next 18 IVLs

**Fig. 3:**
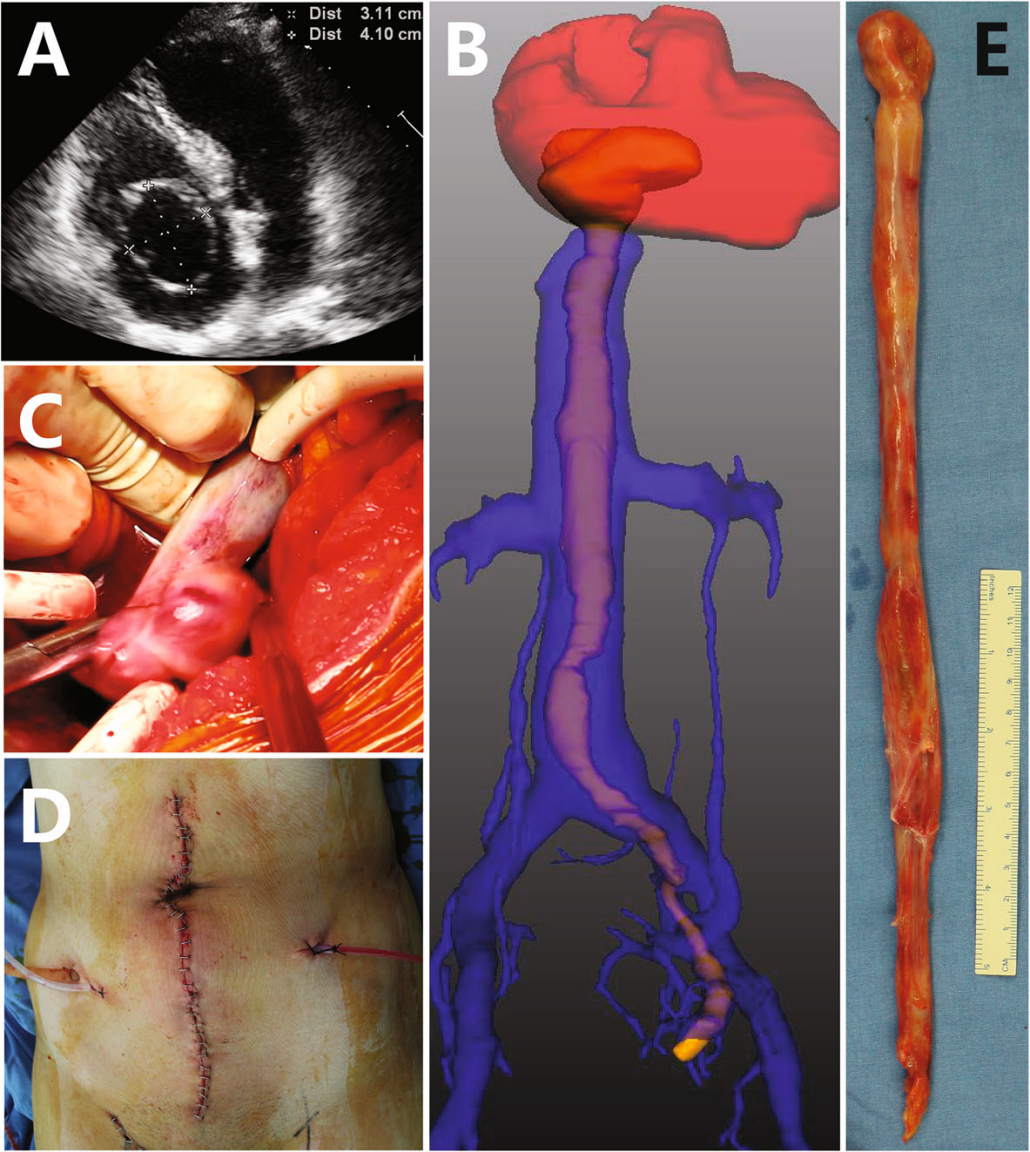
The hollow intracardiac head shown by echocardiogram (**a**) and the intracaval tumor shown by 3D reconstruction of venography (**b**) are extracted from a small venotomy at the left common iliac vein (**c**) through a midline laparotomy (**d**). Pathologic specimen (**e**) shows a shrunken head after thrombectomy

**Fig. 4:**
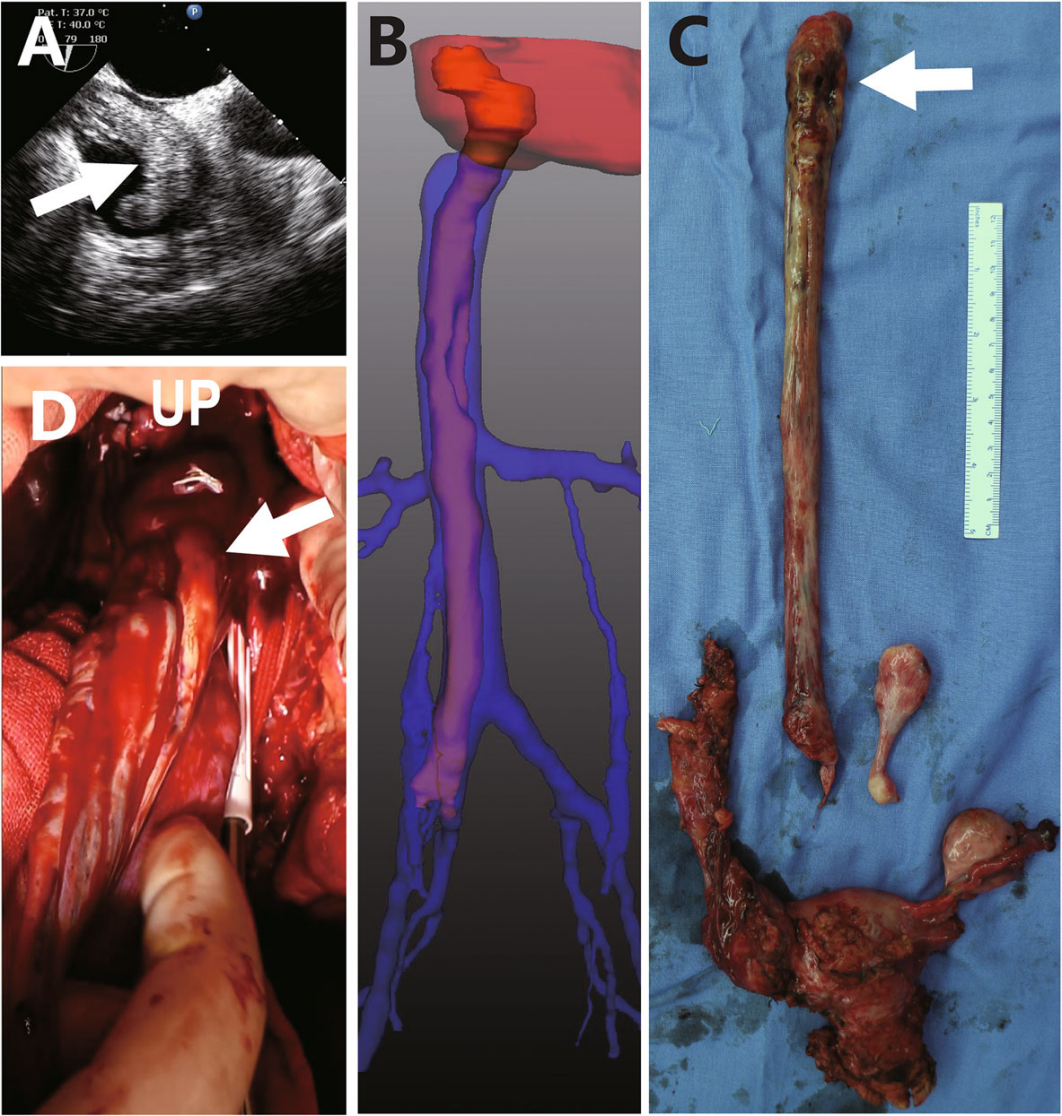
Echocardiogram (**a**) and 3D reconstruction of venography (**b**) indicate that the maximal diameter of intracardiac head is larger than the right common iliac vein. Tumor (**c**) is extracted from an extensive venotomy (**d**) ranging from the supra renal inferior vena cava to the right internal iliac vein with cardiopulmonary bypass. Arrow points to the maximal tumor dilation

**Fig. 5:**
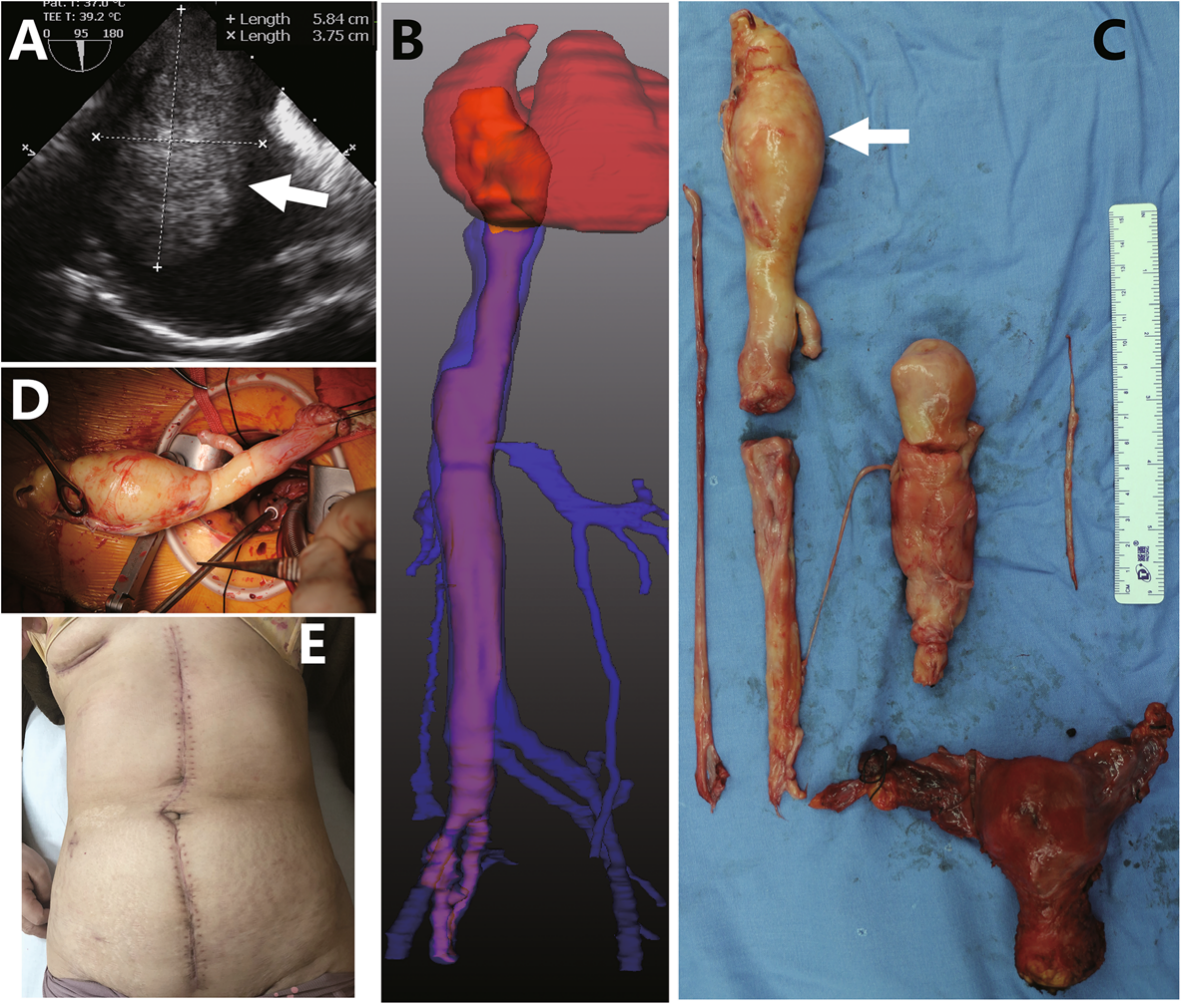
Echocardiogram (**a**) and 3D reconstruction of venography (**b**) indicate that the maximal diameter of intracardiac head is larger than the inlet of inferior vena cava. Tumor (**c**) is dissected through cavotomy and extracted upward through right mini-thoractomy (**d**, **e**). Arrow points to the maximal tumor dilation.

**Fig. 6:**
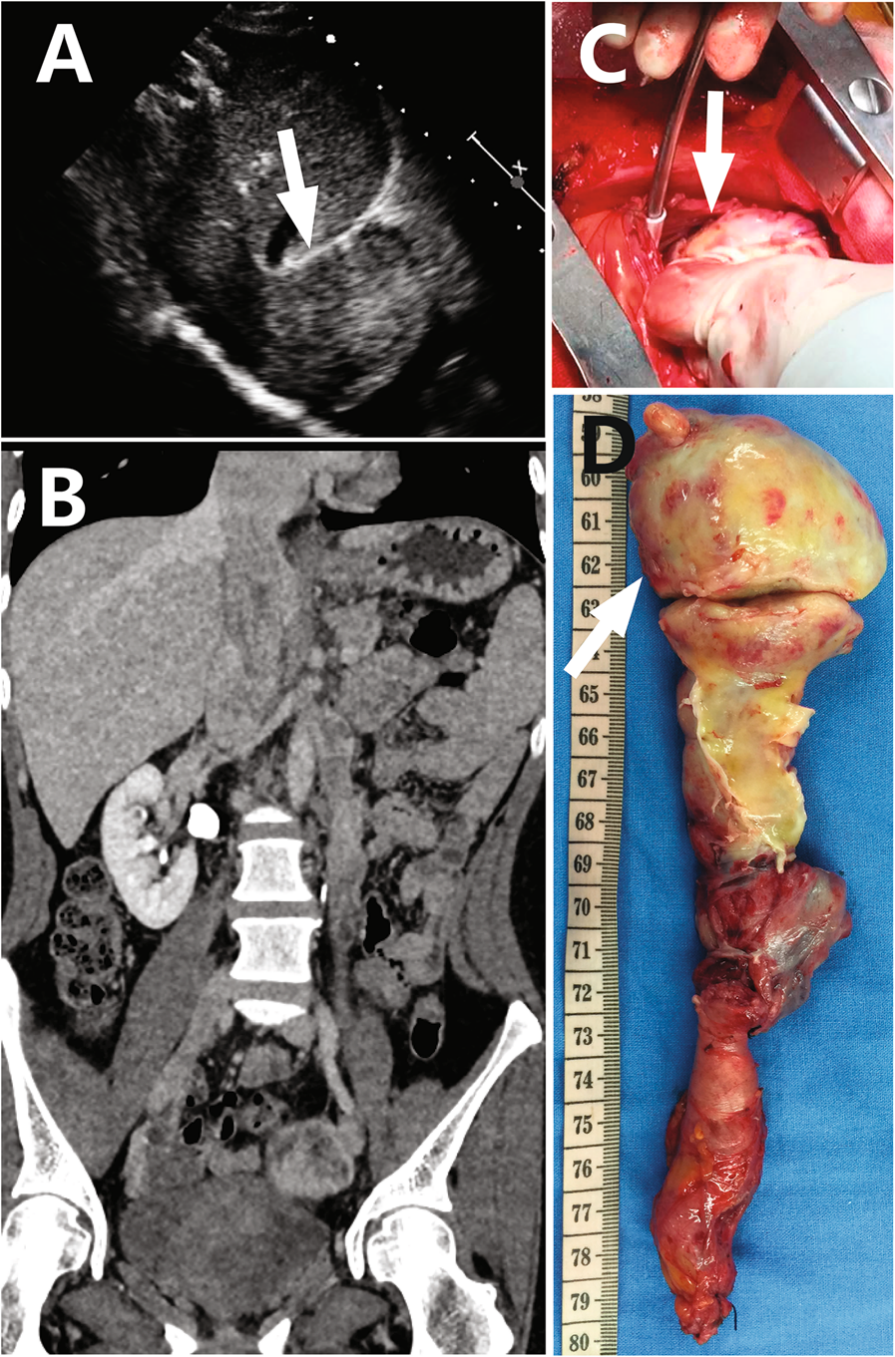
Echocardiogram (**a**) and venography (**b**) indicate that the over-dilated intracardiac head congests the chamber and is immobile. Tumor (**c**, **d**) massively adheres to the junction of the inferior vena cava with the right atrium and is resected through sterno-laparotomy. Arrow points to the site of tumor adherence

Two ICLs (one case shown in Fig. 3) and 4 IVLs confined in IVC received type 1 surgeries, an additional movie file shows the procedure in more detail (see Additional file 1). Seven ICLs (one case shown in Fig. 4) received type 2 surgeries (see more detail in Additional file 2). Among 3 cases of ICLs with over-dilated intracardiac head, one case (Fig. 5) received type 3 surgery (see more detail in Additional file 3). The other 2 cases (one case shown in Fig. 6) received type 4 surgeries because of intracardiac adherence (see more detail in Additional file 4). Tumor adherences with pulmonary artery were presented in the 2 ICLs entering pulmonary artery (one case shown in Additional file 5). Type 4 surgery and pulmonary arteriotomy were performed (see more detail in Additional file 6).

In all the 24 patients, complete removals of tumor portion above the inlet of ovarian vein or internal iliac vein were accomplished. Extra-pelvic circular adherences of tumor were found in 12 (50%) patients (Table 1). In 9 (37.5%) patients, tumors in IVC consisted of multiple (> 1) strands.


Additional file 1 The planning and procedure of type 1 surgery.



Additional file 2 The planning and procedure of type 2 surgery.



Additional file 3 The planning and procedure of type 3 surgery.



Additional file 4 The planning and procedure of type 4 surgery.



Additional file 6 The planning and surgical procedure of tumor extending into pulmonary artery.


### Surgical procedures after tumor Thrombectomy from IVC

If CPB applied, CPB were weaned and protamine administrated, except for 1 case in which CPB continued until the removal of a bulky pelvic intravenous tumor. All patients accomplished bilateral ligations of internal iliac veins and ovarian veins, panhysterectomy and bilateral adnexectomy. Residual pelvic intravenous tumors were found absent in 5 cases, or present in 19 cases including 17 cases achieving macroscopically complete removal by thrombectomy. Two patients with diffused intrapelvic retroperitoneal invasion received incomplete resections due to the high risk of massive hemorrhage. The morphology of tumor thrombus could be bulk (Additional file 7A) or slim string (Additional file 7B).

### Clinical characteristics

For the 18 cases with CPB, the CPB duration ranged from 12 to 132 min (mean, 57.1 ± 46.7 min). As shown in Table 2, intraoperative blood losses, operation time and hospitalization expense in the type 1 surgery group were significantly lesser than the type 2 surgery group and the double-incisions (type 3 surgery and sterno-laparotomy) group. Inpatient stays in the type 1 surgery group were significantly lesser than the double-incisions group. Inpatient stay and hospitalization expense in the type 2 surgery group were significantly lesser than the double-incisions group. There was no evidence of a significant difference between the other pairs.

**Table 2:** Clinical data comparison

	A: type 1 surgery (*N* = 6)	B:type 2 surgery (*N* = 7)	C: double-incisions (*N* = 11)	*P* value (group)	*P* value (A vs B)	*P* value (A vs C)	*P* value (B vs C)
Operation time^a^ (min)	191 ± 31	327 ± 80	395 ± 88	< 0.001	0.004	< 0.001	0.074
Intraoperative blood loss^b^ (ml)	433 ± 186	1200 ± 451	1755 ± 1226	0.004	0.025	0.004	1.000
Inpatient stay^b^ (day)	6.7 ± 0.5	8.1 ± 1.8	13.2 ± 4.9	< 0.001	0.616	0.001	0.042
Hospitalization expense^a^ (RMB)	68,969 ± 7066	94,332 ± 8540	132,693 ± 17,719	< 0.001	0.003	< 0.001	< 0.001

The data are shown as mean ± standard deviation

^a^ equal variance. ^b^unequal variance

Postoperative complications occurred in 4 patients, including thrombosis, coagulation disorders, acute renal failure and fistula of duodenum, each in 1 patient. No significance was found in incidence rate between groups. These patients received effective treatments and recovered well.

### Follow-up

During a mean follow-up of 35.4 ± 27.2 months (cumulative, 70.8 patient-years; range, 1–100 months), all patients were alive, free of recurrences and in NYHA functional class I. In 2 cases receiving incomplete resections of pelvic intravenous tumors, 1 case was treated with Tamoxifen 20 mg daily for 12 months and the other one didn’t receive hormonal therapy, pelvic tumor residues have not changed for 51 and 52 months since operation, respectively. In the three patients with benign metastasizing leiomyoma, the pulmonary nodules remained unchanged 2, 6 and 26 months after operation, respectively.

## Discussion

### The current surgical strategies of extra-pelvic IVL

Literatures focused mainly on developing an adequate surgical strategy [8, 15, 18], but often negated the cost efficiency. Two-stage surgical resection was proven to be effective, but it increased patient burden physiologically and financially. It was true that sterno-laparotomy provided adequate access to the whole path of tumor, however, patients would not be satisfied with such a long incision from suprasternalis to symphysis pubis. Although deep hypothermia circulatory arrest provided a perfectly bloodless field, patients were at risk of related complications such as organ dysfunction, severe coagulation disorder and neurological damage. There were some individual less invasive attempts [23–28] and a few reports [8, 15, 18] have proposed surgical guidelines covering part of these strategies, however, in our opinion, these proposals were not ideal and excluded some patients who would have received less invasive surgery. The present study introduces 4 types of surgical strategies (Fig. 2), and try to propose a more comprehensive guideline (Fig. 1) which can accommodate more patients receiving the less invasive surgery without compromising the curative effect.

### Single laparotomy: tumor Thrombectomy without CPB (type 1 surgery)

A few surgical teams had successfully extracted the freely movable tumor from short venotomy without CPB [18, 23, 25, 26]. If maximal diameter of the proximal tumor is less than or equal to the diameter of vein at the proximal side of short venotomy (Fig. 2a), it is technically feasible to blindly pull out the proximal length of tumor from short venotomy (Figs. 2a and 3c) through the same midline laparotomy as planned panhysterectomy and bilateral adnexectomy. Bleeding can be confidently controlled by vessel loops around the venotomy, therefore CPB can be avoided. Chest-opening is not necessary for ICL. This criterion excludes the possibility of remote tumor adherence. Blunt dissection of adherence at the common iliac vein or the inlet of ovarian vein is confident through these short venotomies. Caution should be taken in pursuing a long incision length of venotomy, due to the risks of excessive bleeding from drainage of lumbar veins and hemodynamic instability by occlusion of the IVC. For such scenario, CPB should be established.

### Single laparotomy: tumor Thrombectomy with CPB (type 2 surgery)

If the maximal diameter of the proximal tumor is larger than the diameter of vein at the proximal side of short venotomy (Fig. 2b), it is technically impossible to drag the tumor out of short venotomy, an extensive venotomy to the site of dilation is needed to release the tumor (Figs. 2b and 4d). In this category, intracaval tumor circular adherence can be problematic. According to a review of 194 patients [7], in 76 patients with adherence situation available, 55 patients developed tumor adherence with intima of IVC or heart, it was in accordance with our cohort (50%). Our observations showed that adherences to the wall of extrapelvic veins only (but not always) occurred at the site of the oversize tumor or near the starting site of tumor afflux (Table 1). The mechanism might be the close contact between the surfaces of tumor and intima under compression stress. Pre-operative assessment should pay attention to possible tumor adherences in the middle of IVC. Recklessly dragging a remote tumor adherence belong the short venotomy is at risk of tearing the IVC, an extensive venotomy to the site of adherence is necessary for blunt dissection. The facilitation of CPB is highly recommended in order to reduce risks of hemodynamic instability and blood loss [24]. In this category, if the maximal diameter of intra-cardiac head of tumor is less than or equal to the diameter of the inlet of IVC (Fig. 2b), the intra-cardiac tumor can still be blindly pulled downward through the inlet of IVC without chest opening.

The upper end of extensive venotomy could be set at the site of the maximal tumor dilation (Fig. 2b), ranging from the infra renal IVC to the supra hepatic IVC. The retro or supra hepatic IVC could be accessed by mobilizing the right hepatic lobe. After pulling out the proximal head of tumor, a serial and sequential cavotomy could be done until the extraction of tumor out of internal iliac vein or ovarian vein. By doing so, the whole length of IVC could be safely opened, any kind of intracaval tumor dilation or adherence at any site of IVC, part of which once was thought to require sternotomy [8, 15, 18], can be resected through laparotomy only.

In order to provide well visualization, deep hypothermia circulatory arrest was often applied by other surgical teams [8, 15, 18, 22]. In our experience, satisfied bloodless field could be obtained by umbilical tapes encircling around the venotomy together with 2 intracardiac suction devices of CPB [24], also by the minimal caval opening during serial and sequential cavotomy. Mild hypothermia was approved to be competent for various types of surgeries in our cohort. Therefore, the adverse effects of circulatory arrest and deep hypothermia could be avoided.

After the implementation of the new guideline, 9 of 14 consecutive ICL cases accomplished single-laparotomy strategy in our cohort, with or without CPB. Also, by review of the first 6 ICLs who received sterno-laparotomies indiscriminately, 4 of them fitted these criteria and could have applied single laparotomy strategy. It is encouraged that our guideline accommodated more patients avoiding chest opening compared with previous proposals [8, 15, 18].

### Double-incisions: mini-thoracotomy (type 3 surgery) or Sternotomy (type 4 surgery)

Once the maximal diameter of intra-cardiac head of tumor is larger than the diameter of the inlet of IVC (Fig. 2c), atriotomy is inevitable. In our experiences, if the tumor head congested the intracardiac cavity and showed signs of immobilization by echocardiography (Fig. 6a) massive tumor adherence could be strongly predicted, sternotomy was neccessary to gain adequate exposure. If not, tumor can be confidently resected through right mini-thoracotomy (Fig. 5d, e). Possible lesion of tricuspid valve [29] could also be repaired or replaced through this minimal incision. Brutal pulling of tumor through atriotomy may tear the intraperitoneal vein [10], the upward removal of tumor can be safer by firstly transecting the tumor through intra-abdominal venotomy (Fig. 2c).

In our cohort, both cases extending into the pulmonary artery developed adherences within pulmonary artery. According to the literatures, adherences inside pulmonary artery might be present [13] or not present [20]. Blind extraction from venotomy inside abdomen seems unsafe for this category of patients, sterno-laparotomy should be prudently planned considering the need of pulmonary arteriotomy.

There are other important rules. Any blind pulling of intracardiac tumor should be under surveillance of transesophageal echocardiography. Surgeons must keep in mind the potential falling tumor debris during extraction. The more radical strategy should always be regarded as a backup plan for any lesser invasive strategy. For example, CPB should be on standby during non-CPB thrombectomy. Single laparotomy should be converted to double-incision if blind extraction of intracardiac portion fails.

### The extent of surgery within pelvic cavity

IVLs sometimes diffusely enter the pelvic venous plexus, radical thrombectomies before any ligations of the ovarian and parametrial venous plexuses are necessary, a meticulous search for tumor thrombus is also needed. Even so, some slim tumor thrombus may hide deeply inside the pelvic venous plexus, which raise uncertainty about whether a complete resection is accomplished. Therefore, prophylactic bilateral ligations of internal iliac vein and ovarian vein are imperative to prevent potential recurrence entering the IVC [30, 31].

### Hormonal therapy

In some reports [16, 32, 33], hormonal therapies were effective to prevent recurrence after incomplete resections. In our cohort, tumor did not recur for almost 4 years each in the 2 cases following incomplete resections of pelvic intravenous tumor, while hormonal therapy was absent or given for a short period of 1 year. The role of hormonal therapy is still controversial [7, 17].

### Study limitations

This study is a retrospective analysis, the small number of patients analyzed and various confounding factors limits the statistical power. Because the proposed guideline was implemented just 4 years ago in our institution, there is lack of data on long term follow-ups (> 4 years) after less invasive operations. Because of the extreme low incidence of this disease, in our cohort, there was only individual or no case fit for some criteria of the proposed guide which was designed to include all possible forms of extra-pelvic IVLs. More cases and longer follow-ups are needed in future study to validate the utility of the proposed guide.

## Conclusions

For various extra-pelvic IVLs, the 4 types of surgical strategies including less invasive options are feasible, providing these are selected by a guideline base on the tumor extension and morphology. The non-CPB and single-laparotomy surgical strategies are more cost-effective. The proposed guideline of pre-operative planning is believed to accommodate more patients avoiding sternotomy or CPB without compromising the curative effect.

## Supplementary information

**Additional file 5.** Tumor extending into pulmonary artery receives type 4 surgery.

**Additional file 7.** Different shapes of pelvic intravenous tumors.

## Data Availability

All data generated or analyzed during this study are included in this published article.

## Abbreviations

CPB: Cardiopulmonary bypass
ICL: Intracardiac leiomyomatosis
IVC: Inferior vena cava
IVL: Intravenous leiomyomatosis

## References

[1] Carr RJ, Hui P, Buza N (2015). Intravenous leiomyomatosis revisited: an experience of 14 cases at a single medical center. Int J Gynecol Pathol.

[2] Merchant S, Malpica A, Deavers MT (2002). Vessels within vessels in the myometrium. Am J Surg Pathol.

[3] Norris HJ, Parmley T (1975). Mesenchymal tumors of uterus. V. Intravenous leiomyomatosis - a clinical and pathologic study of 14 cases. Cancer.

[4] Ordulu Z, Nucci MR, Dal Cin P (2016). Intravenous leiomyomatosis: an unusual intermediate between benign and malignant uterine smooth muscle tumors. Mod Pathol.

[5] Buza N, Xu F, Wu W (2014). Recurrent chromosomal aberrations in intravenous leiomyomatosis of the uterus: high-resolution array comparative genomic hybridization study. Hum Pathol.

[6] Durck H (1907). Ueber ein kontinvierlich durch die learned Holvene in das Herz vorwachsendes Fibromyom des uterus. München Med Wochenschr.

[7] Li B, Chen X, Chu YD (2013). Intracardiac leiomyomatosis: a comprehensive analysis of 194 cases. Interact Cardiovasc Thorac Surg.

[8] Ma G, Miao Q, Liu X (2016). Different surgical strategies of patients with intravenous leiomyomatosis. Medicine (Baltimore).

[9] Kuenen B, Slee P, Seldenrijk C, Wagenaar S (1996). Intravenous leiomyomatosis complicated by Budd—Chiari syndrome. Postgrad Med J.

[10] Nili M, Liban E, Levy MJ (1982). TRICUSPID STENOSIS DUE To intravenous LEIOMYOMATOSIS - a CALL for caution - CASE-report and review of the literature. Tex Heart Inst J.

[11] Roman DA, Mirchandani H (1987). Intravenous leiomyoma with intracardiac extension causing sudden death. Arch Pathol Lab Med.

[12] Marcus SG, Krauss T, Freedberg RS (1994). Pulmonary embolectomy for intravenous uterine leiomyomatosis. Am Heart J.

[13] Rajaii-Khorasani A, Kahrom M, Hashemzadeh M (2012). Pulmonary artery extension of uterine leiomyoma. J Card Surg.

[14] Burke M, Opeskin K (2004). Death due to intravenous leiomyomatosis extending to the right pulmonary artery. Pathology.

[15] Gan HL, Zhang JQ, Bo P (2009). The classification and surgical strategy of intracardiac leiomyomatosis. Asian J Surg.

[16] Grella L, Arnold TE, Kvilekval KHV, Giron F (1994). Intravenous leiomyomatosis. J Vasc Surg.

[17] Doyle MP, Li A, Villanueva CI (2015). Treatment of intravenous Leiomyomatosis with cardiac extension following incomplete resection. Int J Vasc Med.

[18] Gan HL, Zhang JQ, Zhou QW (2011). Surgical treatment of intracardiac leiomyomatosis. J Thorac Cardiovasc Surg.

[19] Ariza A, Cerra C, Hahn IS, Shaw RK, Rigney B (1982). Intravascular leiomyomatosis of the uterus. A case report. Conn Med.

[20] Ricci MA, Cloutier LM, Mount S, Welander C, Leavitt BJ (1995). Intravenous leiomyomatosis with intracardiac extension. Cardiovasc Surg.

[21] Price JD, Anagnostopoulos C, Benvenisty A, Kothuru RK, Balaram SK (2017). Intracardiac extension of intravenous Leiomyomatosis. Ann Thorac Surg.

[22] Rispoli P, Santovito D, Tallia C (2010). A one-stage approach to the treatment of intravenous leiomyomatosis extending to the right heart. J Vasc Surg.

[23] Subramaniam B, Pawlowski J, Gross BA, Kim YB, LoGerfo FW (2006). TEE-guided one-stage excision of intravenous leiomyomatosis with cardiac extension through an abdominal approach. J Cardiothorac Vasc Anesth.

[24] Li H, Xu D, Lu W, Wang C (2016). Complete resection of intracardiac leiomyomatosis through an abdominal approach under peripheral cardiopulmonary bypass. J Thorac Cardiovasc Surg.

[25] Harris LM, Karakousis CP (2000). Intravenous leiomyomatosis with cardiac extension: tumor thrombectomy through an abdominal approach. J Vasc Surg.

[26] Matsuo K, Fleischman F, Ghattas CS (2012). Successful extraction of cardiac-extending intravenous leiomyomatosis through gonadal vein. Fertil Steril.

[27] Schindler N, Babrowski T, DeSai T, Alexander JC (2012). Resection of intracaval leiomyomatosis using abdominal approach and venovenous bypass. Ann Vasc Surg.

[28] Jeanmart H, Lecompte P, Casselman F (2006). Endoscopic tumor resection of the inferior vena cava. J Thorac Cardiovasc Surg.

[29] Castelli P, Caronno R, Piffaretti G, Tozzi M (2006). Intravenous uterine Leiomyomatosis with right heart extension: successful two-stage surgical removal. Ann Vasc Surg.

[30] Gehr NR, Lund O, Alstrup P (1999). Recurrence of uterine intravenous leiomyomatosis with intracardiac extension. Diagnostic considerations and surgical removal. Scand Cardiovasc J.

[31] Mazzola A, Gregorini R, Procaccini B (1986). Intracaval and intracardiac leiomyomatosis of uterine origin. Ann Vasc Surg.

[32] Biri A, Korucuoglu U, Zumrutbas N, Tiras B, Guner H (2008). Intravenous leiomyomatosis treated with aromatase inhibitor therapy. Int J Gynecol Obstet.

[33] Mitsuhashi A, Nagai Y, Sugita M, Nakajima N, Sekiya S (1999). GnRH agonist for intravenous leiomyomatosis with cardiac extension. A case report. J Reprod Med.

