# Supplementary figures and images for: Surgical treatment strategies for extra-pelvic intravenous leiomyomatosis
Source: Orphanet J Rare Dis. 2020 Jun 16;15:153. doi: 10.1186/s13023-020-01394-9 (PMC7296750; doi:10.1186/s13023-020-01394-9)

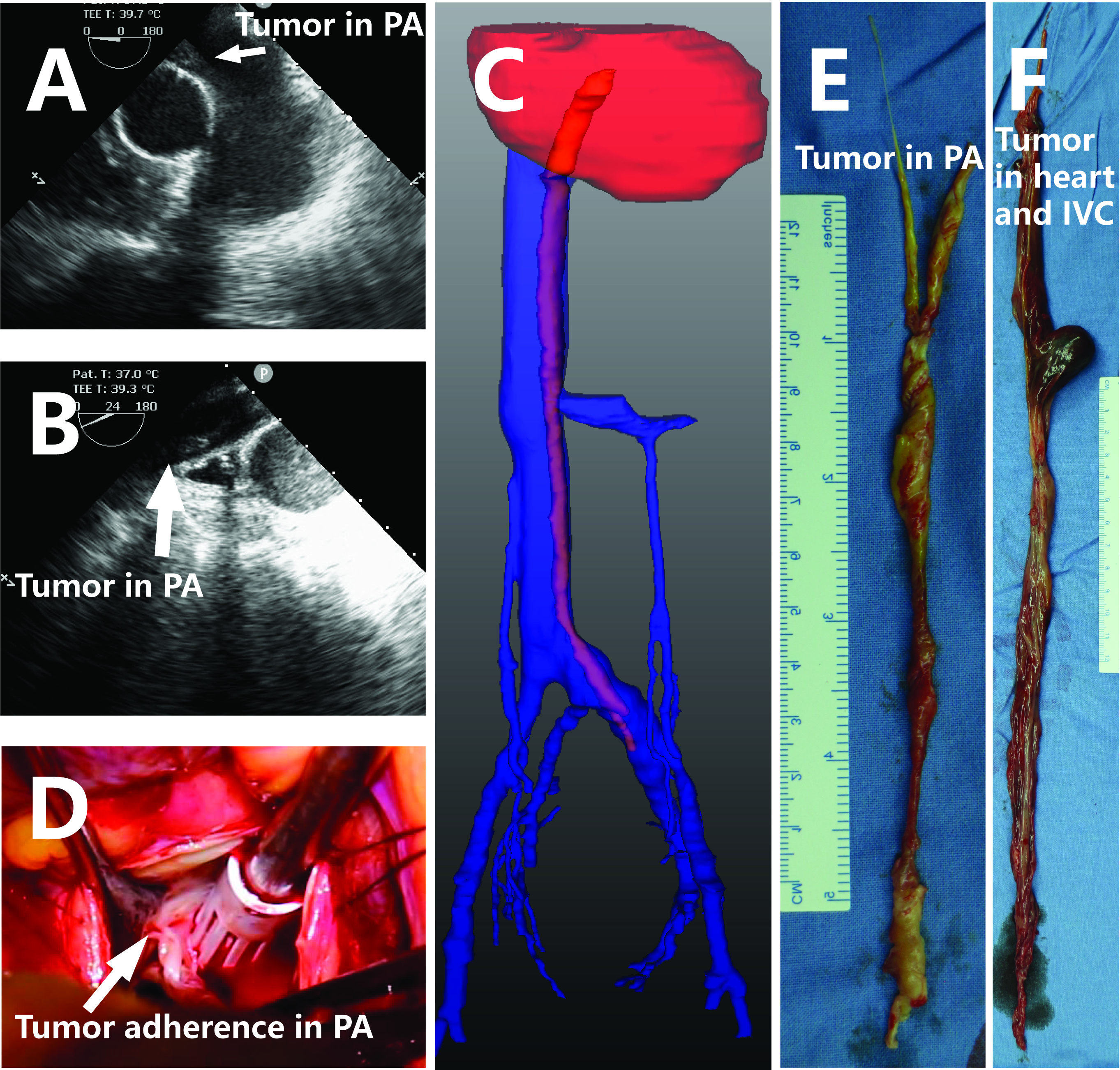

Supplement: Supplementary file 6 — Additional file 5. Tumor extending into pulmonary artery receives type 4 surgery. [file 13023_2020_1394_MOESM5_ESM.tif]

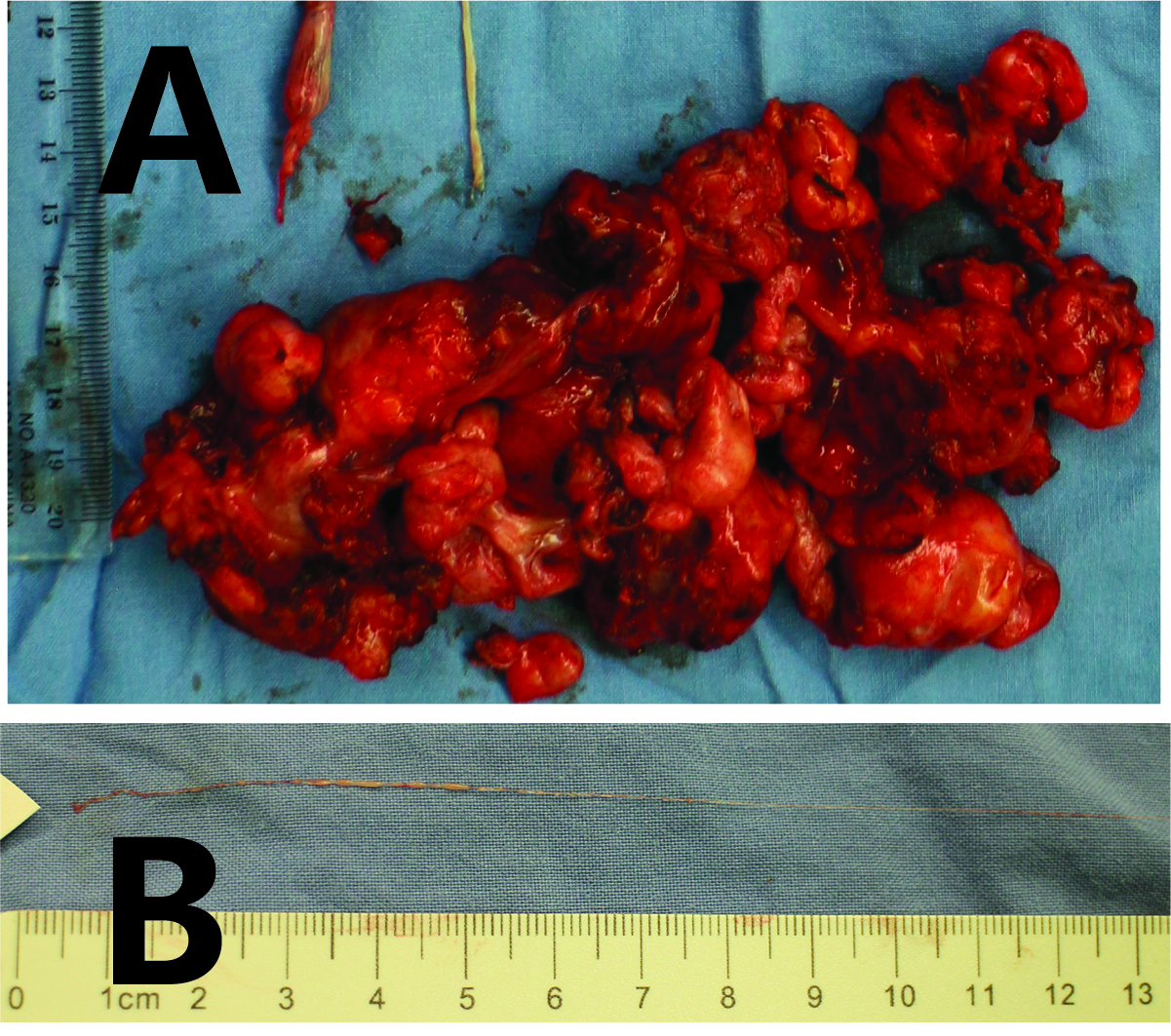

Supplement: Supplementary file 7 — Additional file 7. Different shapes of pelvic intravenous tumors. [file 13023_2020_1394_MOESM7_ESM.tif]
